# Changing the food environment in secondary school canteens to promote healthy dietary choices: a qualitative study with school caterers

**DOI:** 10.1186/s12889-024-19513-7

**Published:** 2024-07-23

**Authors:** Marie Murphy, Alice Coffey, Miranda Pallan, Oyinlola Oyebode

**Affiliations:** 1 Institute of Applied Health Research, University Birmingham, Birmingham, UK; 2 Institute of Global Sustainable Development, University of Warwick, Coventry, UK; 3 Wolfson Institute of Population Health, Queen Mary University of London, London, UK

**Keywords:** Adolescents, Diet, Healthy eating, Schools, Catering, Choice architecture, Qualitative

## Abstract

**Background:**

Typical adolescent diets do not meet current dietary recommendations. There is a need to address these dietary patterns to reduce the risk of obesity and other diet-related diseases. Schools provide an opportune setting to do so, as students consume a substantial proportion of their daily dietary intake whilst at school. There is a developing evidence base on the use of choice architecture (food choice cues) to promote healthy eating in school contexts. It is necessary to understand the acceptability and feasibility of implementing such interventions. We aimed to explore these factors from the perspectives of secondary school caterers.

**Methods:**

We conducted qualitative interviews with caterers from secondary schools across the West Midlands, UK and national/regional catering representatives. A semi-structured topic guide and visual aid were used to guide interviews. Interviews were recorded and transcribed. Framework analysis was conducted in NVivo v12.

**Results:**

Twelve participants took part. Seven themes were identified and grouped into three categories: Acceptability (Suitability; Salient cues; Student engagement), Barriers (Catering decision drivers; Limits of influence), and Enablers (Perceived role; Opportunities). Caterers considered healthy food cues to be suited to adolescents as they require minimal reflective motivation. Salient cues included enhancing the placement, presentation and portability of healthy items, improving the dining environment and focusing pricing/incentive strategies on increased quantity. Student engagement was considered important. Some catering decision drivers conflicted with healthy food cues, and many felt that their role in healthy eating was limited due to the overwhelming influence of external food environments, adolescent resistance, and features of the secondary school canteen setting e.g. short duration of lunchtime, lack of space. However, caterers appeared motivated to implement healthy food cues and identified key opportunities for implementation, including integration into whole-school approaches to healthy eating.

**Conclusions:**

Interventions using healthy food cues appeared acceptable to secondary school caterers, key potential implementers of these strategies. Future interventions could incorporate strategies relating to placement, presentation and pricing to prompt healthy selections, and actions to engage the student body and improve the dining environment. Evaluations should consider potential impacts upon food purchasing, consumption and waste to address caterers’ concerns about these issues.

**Supplementary Information:**

The online version contains supplementary material available at 10.1186/s12889-024-19513-7.

## Background

As a population group, the dietary patterns of adolescents are poor [1] and obesity in children and adolescents has risen substantially in most high-income countries over the past three decades [2, 3]. In the UK, the National Diet and Nutrition Survey [4] shows that intakes of free sugars were at 12.3% of total energy intake in children aged 11–18 years, with only 7% meeting the recommended ≤ 5% of total energy intake, and the largest contributors being sugar sweetened beverages (SSBs). Only 4% of 11–18 year olds meet UK fibre recommendations and 12% meet the recommendations for five fruits and vegetables per day, averaging 2.9 portions. Saturated fat intakes were higher than recommended across all age groups. Overweight and obesity are currently highly prevalent in British children, with 23.4% classified as obese and 14.3% as overweight the year prior to entering secondary school (10–11 years) [5]. On this basis, there is a need to address the poor dietary patterns of adolescents in the UK to reduce their risk of obesity and other diet-related diseases, during both adolescence and later in life.

Schools provide an opportune setting in which to target the dietary behaviours of adolescents, as children spend a substantial proportion of their time at school and typically at least one meal a day is consumed on the school site. Health and wellbeing is widely viewed as part of the overall educational remit of schools [6], and schools are considered to play a significant role in promoting healthy eating in children and adolescents through interventions relating to the physical environment, education, and food policies and provision [7–9]. However, there is lack of consistent, high-quality evidence on the most effective approaches for improving dietary intakes in secondary school aged-pupils [8, 10]. In England, schools have a legal duty to provide food to pupils, and school food standards are in place to ensure the nutritional quality of this provision [11], but there is evidence of poor implementation and limited impact in the secondary school context [12, 13], indicating that additional support for healthy eating, such as strategies to guide healthy food and drink selection, may be required. In most schools, pupils also have the option to bring in a packed lunch, and some secondary schools may allow pupils off-site at lunchtime. In secondary schools, most schools provide food in a canteen-style format, with meals typically prepared on-site, and a range of hot and cold options available to purchase. Around a quarter of secondary school pupils in England are eligible for means-tested Free School Meals [14].

Choice architecture (CA) may prove an effective means of changing dietary behaviours in adolescents in UK secondary schools given its effectiveness in other school and university settings [15–18]. CA is defined by Hollands et al. as *“those [interventions] that involve altering small-scale physical and social environments*,* or micro-environments to cue healthier behaviour”* [19]. CA is an aspect of nudge theory [20], an approach to improving health behaviour by influencing automatic or non-conscious psychological processes [21]. The proposed advantage of nudge is that it is a behaviour change approach that does not rely on conscious motivation, so requires little or no cognitive engagement; whilst traditional health promotion interventions target reflective, goal-oriented psychological processes, requiring individuals to make use of cognitive capacity [21]. This approach may suit adolescents, as it retains freedom of choice at an age where independence in decision-making is given high priority [22]. The Typology for Interventions in Proximal Physical Environments [23], provides a framework for characterising CA, with examples including altering the properties and/or placement of objects or stimuli [19]. CA could be used to promote many of the healthy dietary behaviours that are currently suboptimal in the adolescent population - for example to increase consumption of fruit and vegetables and fibre or to reduce consumption of foods high in fat, salt or sugar. A meta-analysis of the effect of CA interventions upon fruit and vegetable behaviours [24] found that such interventions had a moderately significant effect (*d* = 0.30) upon increasing fruit and/or vegetable choice and sales or servings, with the largest effect seen for altering the placement of food items (*d* = 0.39) and combined strategies (*d* = 0.28).

Problems of acceptability, compliance and delivery of the intervention often undermine the success of intervention evaluations [25]. In developing interventions, understanding attitudes towards the intervention and the contexts in which they take place, are important in understanding the theoretical basis for success (or lack of success) [25]. In this setting, the views of school catering teams and providers towards the intervention are crucial as this group would be largely responsible for implementation. A recent policy brief by the World Health Organization (WHO) on using nudges in schools to promote healthy eating highlights the importance of engagement and empowerment of food service staff for implementation success [26]. Research in the US found that in schools implementing CA strategies, there was a positive correlation between catering managers’ support of CA strategies and the extent of strategy use [27]. Gathering the views of catering providers prior to intervention design is therefore needed to understand acceptability, feasibility, cost-effectiveness and potential opportunities and challenges. This information can be incorporated into the planning stage when developing future interventions.

## Methods

### Aims/objectives

We aimed to collect data from school catering teams to inform the development of future food choice interventions. The objectives were to explore secondary school catering team/provider views on (1) the potential acceptability and feasibility of implementing specific food choice cues in their canteen; and (2) the potential barriers to implementation.

### Study design, setting, sampling and recruitment

This was a qualitative study with catering representatives in state-funded secondary schools in the West Midlands, UK. Ethical approval was granted by the University of Birmingham’s Science, Technology, Engineering and Mathematics Ethical Review Committee on the 25th May 2021 (ERN_21–0514). Participants provided written informed consent to participate. The study has been reported according to the COREQ checklist.

We aimed to recruit a minimum of 10 schools/catering providers with a range of catering provision models (school-employed catering staff versus external catering contractors) and with variation in the proportion of pupils eligible for free school meals (%FSM) within the school sample. This purposive sampling approach was intended to support exploration of the potential influence of the sociodemographic characteristics of pupils and the catering arrangements upon views towards implementing food choice cues in secondary schools, and to address weaknesses in previous research in which qualitative research on the topic of school food choice in the UK has been conducted in a small number of sites [28, 29]. Eligible schools in our target areas (Birmingham, Coventry, Dudley, Sandwell, Solihull and Warwickshire; *n* = 196) were identified through national school census data and split into two groups based on being above (*n* = 110) or below (*n* = 86) the mean %FSM for area [30]. The two groups were randomly ordered, and schools were invited sequentially between June 2021 and January 2022 in a phased approach (*n* = 117 schools had been invited by the end of the recruitment period).

Following study commencement, we contacted/invited the catering manager/staff within these schools by email/phone, aiming for 1–2 staff per school. We also invited representatives from regional or national catering providers involved in planning menus or working directly with schools in the area (*n* = 10). These providers were identified via prior research in these settings [13] and through searching webpages of schools within the sampling frame. Potential participants were invited by email, sent a participant information sheet in advance (which included details about the purpose of the study) and provided written informed consent. Participants received a £20 shopping voucher as a thank you for their time.

### Data collection

One-to-one video interviews (Zoom Video Communications Inc) were selected to minimise participant burden and maximise recruitment, and to ensure the safety of researchers and participants during the COVID-19 pandemic. Phone interviews were also offered for those with limited computer/internet access or if preferred. Participants completed a short demographic questionnaire collecting information about their role and organisation. Interviews were carried out by two female researchers, one experienced qualitative researcher (MM) and one pre-doctoral researcher (AC) using a semi-structured topic guide. A visual aid, comprising images and brief explanations of 33 selected food choice cues was sent to participants in advance and used during the interview to support discussion. Both the topic guide (additional file 1) and visual aid (see Table 1) were developed from previous research conducted on this topic [28, 31–34] and selected CA strategies for inclusion were based on an existing framework [23]. Interviews were recorded with written consent using a digital audio recorder and were transcribed verbatim by an external transcription service.

**Table 1:** Framework for selection of strategies featured in visual aid and example strategies

Strategy type^1^	Examples of strategies^2^
Availability	• Increasing the variety of fruits and vegetables available
Positioning	• Positioning fruit at multiple places in the line • Cakes, biscuits and desserts to be staff-served only
Functionality	• Increasing the portability of the healthiest options
Presentation	• Pre-chopped fruits; attractive presentation of healthiest dishes • Dining room decoration to improve ambience
Information	• Simple labelling; rich description for healthy dishes • Promotional posters to prompt healthy choices
Pricing / promotions	• Meal deal options and loyalty cards
Decision structure / assistance	• Pre-ordering; verbal prompts / upselling fruit / water at till • Defaults e.g. serving of vegetables with all dishes
Participatory approaches	• Involving students in naming products / creating dishes

^1^Strategy types were based on an existing CA framework [23]; ^2^Examples were based on previous research conducted on this topic [28, 31–34].

### Analysis

Data were analysed using the framework method, a systematic approach to qualitative thematic analysis that was considered appropriate given its extensive application in research teams where there is a range of experience in qualitative analysis [35]. Three researchers (MM, AC, OO) were involved in coding transcripts in NVivo v12 [36] as follows: (1) independent free-coding of a sample of transcripts using an inductive approach; (2) agreement of a coding framework; (3) application of coding framework to all transcripts. A framework matrix was produced, summarising the data by each code for each transcript, and reviewed by the team to explore potential connections and themes within the data. This analysis was guided by social constructivist orientations [37], aimed at exploring a range of participant perspectives to construct a broad, interpretive understanding of the acceptability and feasibility of CA strategies and barriers to their implementation, as outlined in the aims/objectives. Pseudonymised direct quotes have been used to illustrate themes.

## Results

### Sample description

We recruited 12 participants, comprising nine catering staff from eight different schools (response rate = 7%) and three catering representatives from two regional/national catering companies (response rate = 20%). In six of the eight schools, the proportion of pupils eligible for FSM was above the England average of 18.9% (2020/2021) [38] and in four of these schools %FSM eligibility was above the average for area (3.6% of schools in sampling frame) whilst four had below average %FSM for the area (4.6% of schools in sampling frame). There was an equal split of in-house and external school catering teams. Interview duration ranged from 27 to 78 min, with a mean length of 48 min. Participant characteristics are displayed in Table 2 and school characteristics in Table 3.

**Table 2:** Participant characteristics

ID	Job Title	Organisation ID number	Years at organisation	Years in school catering
1	Catering Supervisor	S^1^1	11–15 years	More than 15 years
2	Catering Manager	S2	1–2 years	6–10 years
3	Catering Manager	S4	More than 15 years	More than 15 years
4	Supervisor and Cook	S2	More than 15 years	More than 15 years
5	Operations Manager	C^2^2	6–10 years	More than 15 years
6	Company Nutritionist	C2	6–10 years	6–10 years
7	Operations Manager	C1	11–15 years	More than 15 years
8	Catering Manager	S3	11–15 years	11–15 years
9	Catering Manager	S6	1–2 years	6–10 years
10	Catering Manager	S5	More than 15 years	More than 15 years
11	Catering Manager	S7	More than 15 years	More than 15 years
12	Catering Manager	S8	More than 15 years	More than 15 years

^1^S=school; ^2^C=Catering provider

**Table 3:** School characteristics

ID	Participants	Catering provision^1^	% FSM^2^	Above/below mean for area^3^	Above/below mean for England
S1	1	External	19%	Below	Above
S2	2	External	13%	Above	Below
S3	1	Inhouse	31%	Above	Above
S4	1	Inhouse	21%	Below	Above
S5	1	Inhouse	15%	Below	Below
S6	1	External	38%	Above	Above
S7	1	Inhouse	30%	Below	Above
S8	1	External	31%	Above	Above
C1	1	Catering provider	n/a	n/a	n/a
C2	2	Catering provider	n/a	n/a	n/a

^1^Inhouse = school-employed catering team; External = external catering contractor

^2^Percentages rounded to minimise risk of identification. ^3^Local Authority

### Thematic analysis

In total, 66 codes were used to describe the data. Seven themes were identified and grouped into three categories: Acceptability (Suitability; Salient cues; Student engagement), Barriers (Catering decision drivers; Limits of influence), and Enablers (Perceived role; Opportunities). Sub-themes were identified within several themes. Theme/sub-theme descriptions and illustrative quotes are provided below. A summary table of categories and themes is provided (Table 4).

**Table 4:** Summary table of categories and themes

Category	Theme	Summary description
Acceptability	*Suitability*	Use of healthy food cues was seen to be a viable approach in this population and setting as they require minimal reflective motivation.
	*Salient cues*	The most salient cues were those relating to placement, visual appeal, portability and pricing/promotion of foods, and improvements to the dining environment.
	*Student engagement*	Student engagement was seen as important in designing appealing menus that meet students’ needs.
Barriers	*Catering decision drivers*	There were concerns over the potential for increased waste and loss of custom through greater use of healthy food cues, and the negative impact that this would have upon financial viability of the service. Adherence to legal frameworks/concerns around food allergies also created barriers to implementing some strategies.
	*Limits of influence*	Caterers considered their role in healthy eating as limited due to the overwhelming influence of external food environments, practical constraints on their ability to implement some strategies, and adolescent resistance.
Enablers	*Perceived role*	Caterers saw themselves as having a role in influencing adolescent dietary intake and eating behaviours.
	*Opportunities*	Caterers supported early intervention, integrating healthy school food provision into whole-school approaches, and using an adaptable, responsive approach.

#### Acceptability

***Theme 1: Suitability***

Caterers considered CA to be suited to adolescents as it requires minimal reflective motivation on the part on the young person and maintains choice without pupils feeling ‘forced’ upon, a factor considered important during adolescence.

“I think if you tell somebody to eat healthier or this is healthier for you, I think at that age they don’t necessarily… that’s not a motivating factor almost. So I do think nudging their environments to make it… just by default I do think that’s… it’s a sneaky way but I do think you might have a better uptake on that rather than just saying this is healthier, because at that age you know what’s healthier but they are obviously not choosing it. So sometimes information alone, knowledge alone isn’t enough to stop or to promote you doing something.” ID5.

***Theme 2: Salient cues***

Sub-theme 2a: Placement

Strategies that focus on positioning fruits and water in convenient places, at multiple places in the food service area, at eye-level and/or near to the till were seen as effective in increasing purchases of these items as it provided a visual cue for selection and provided an opportunity to ‘upsell’.

However, it was apparent that less healthy items, such as cakes, biscuits and pastries were also available at the till, so could provide direct competition to the purchasing of fruit/water.

“…our fruit bowl is by the till, and whatever chopped fruit like melon slices etc., but they usually are near the till point anyway, as are all the cakes as well” ID10.

Defaults, such as including salad in sandwiches and serving a portion of vegetables with main meals as standard were also seen to be acceptable and successful, in the experience of some participants.

Sub-theme 2b: Visual appeal

The visual appeal of food was seen as a key element in promoting the selection of more favourable items such as main meals, vegetables and salads. As well as presentation of colourful, vibrant dishes, this also extended to attractive packaging or plating and counter displays.

Likewise, participants felt that enhancing the visual appeal of the eating environment (e.g. student artwork, attractive décor, posters) would enhance uptake of school meals and encourage selection of sit-down meals (as opposed to “grab-and-go”). This would create an environment which encourages sociability and the knock-on effects would be that students stay and eat for longer and are encouraged to eat a more balanced meal.

“Yeah, if you’ve got more space to sit down then you’re more likely to choose something like a main meal potentially… But if it’s a place where a student wants to be then you’re more likely to get more engagement and potentially healthier food eating, potentially.” ID5.

However, participants perceived that schools also need to address the volume of pupils using the eating space if an attractive dining room is to have an impact on uptake of sit-down meals.

“Although you try to make them a nice space for them to sit and have lunch it’s so busy in there, and noisy with so many children, they particularly want to go outside” ID11.

Sub-theme 2c: Grab-and-go appeal

Conflicting with this was a perception of high demand for “grab-and-go” foods that mimic high-street offerings, so a counter strategy proposed was to enhance the portability of main meals; or to make grab-and-go items more nutritionally complete, rather than to try and force pupils to have a sit-down meal. This was seen to better meet pupil preferences.

“They very much like the whole grab and go concept, that’s a thing, they just want to rush out, go and play football, go and sit in a corner and chat to their friends” ID3.

Sub-theme 2d: Pricing/promotion strategies

Pricing/promotion strategies were seen as very influential upon student food and drink selections, and the most effective examples were believed to be those that focused on getting increased value for money i.e. “extra” or free meal elements, meal deals, loyalty cards.

“All of our meal deals which we do we do try and put a free item in there and make it look like it actually… say well they’re always making a saving for choosing that healthier option. But we like to put like yes you have got the choice of a fruit pot or salad or something to go with that.” ID7.

***Theme 3: Student engagement***

Student engagement was seen as important, particularly in gathering feedback on what students like, so that an appealing menu can be designed. This included consulting with Student Councils and pupil surveys, as well as informal feedback in situ. Responding to students’ likes/dislikes was seen as way to boost sales but also support students in decision-making. This also provided a way to better meet the needs of students e.g. Halal options.

“It’s just a continuing engagement piece, and you are continually having… seeing how that feedback you get from students and customers affects your service, and how you can adapt it and make it better.” ID6.

“Have a chat to the kids and you can get feedback from them, you can get them to taste it, and once one of the crowd tastes it then you will get the others, again peer pressure, they will come over and taste it. You just… and we will go with that feedback” ID11.

Other types of student engagement included formal strategies such as taste tests, competitions and workshops. These were seen as promotional activities to engage students in their canteen and in school food.

“I have involved the students previously actually coming up with dishes, recipes, and have cooked with some of the students in the kitchen, for them to actually put the dish out that they have chosen, the recipe that they have come up with, which was good, it was a nice experience for us to have the kids in the kitchen, and also a nice experience for them to see how our kitchen runs and how hard it is for us to do all that we do” ID11.

#### Barriers

***Theme 4: Catering decision drivers***

Sub-theme 4a: Negative impacts on business

Some catering decision drivers conflicted with healthy food cues. Caterers were constrained by a perception that some of these strategies have potentially negative impacts on business, with associated costs and impacts upon custom, waste and profitability. Popular items such as cake, pizza, etc. were seen as large income-generators and were quite cheap to make, and caterers were reliant on these to some extent to achieve a profit/break-even. Caterers were also concerned that offering more healthy items and prohibiting sales of less healthy items would lead to a loss of custom, as pupils would buy their food elsewhere or bring it from home.

“I think one of the things is chocolate is always going to sell, and so I look at it [healthy vending machine options] from my nutritionist head and I am like that’s a brilliant idea, you look at it from a business head and you’re like maybe… it’s really difficult to get the balance right.” ID6.

There were also concerns about high levels of kitchen waste with increasing the volume of fruit/vegetables/main meal sales, as caterers believed that there would be low uptake by pupils.

“Having to throw it away, and with the constraint with budget being so tight we can’t afford to waste food really.” ID3.

Staff were concerned that some strategies, such as moving from takeaway packaging to plated meals, would increase queue sizes/waiting times.

In-house caterers appeared to be more willing to absorb some of the additional costs associated with healthier foods and some of these strategies.

“…we’re not a profit-making organisation, we work for the school, we don’t work for a private company, so as long as we cover our costs and we’re not running a debt, I think that’s all we aim to do really. We aim to just be a break-even service.” ID11.

Sub-theme 4b: Concerns about food allergies

Caterers stated how changing menus to incorporate new dishes/ingredients/items was not straightforward due to concerns about food allergies and a requirement to re-label dishes and avoid cross-contamination in the kitchen.

“Since Natasha’s law [requiring pre-packed directly-for-sale products to be labelled with a full list of ingredients] we have been quite central in our approach, so every single school will produce the same ham sandwich in terms of the products. We have not given it… you could have it so that each individual site was in control of their own labels, but that would just be a nightmare for us because we’ve got so many sites. So we have gone down a route of everything is spec’d out now, so a ham sandwich, this is the label for it and you can only buy these ingredients. So adding more products to that will just take a bit of time.” ID5.

Introducing attractive packing or quick-reference nutrition labelling for certain items was also seen as problematic as many caterers will not pre-package foods as these items would then require additional scrutiny under Natasha’s Law. Adding nutritional labels to items was also seen as time-consuming and overwhelming alongside these legal requirements.

“Because of Natasha’s Law us as a company have gone down the line of we do not prepare our own [pre-packaged directly-for-sale] foods at all, because of the allergen information, so we don’t do any pre-packed items at all, no, so we don’t use any labels anymore.” ID7.

***Theme 5: Limits of influence***

Sub-theme 5a: Wider influences upon adolescent diets

Caterers believed there are other external factors outside of the school environment that influence adolescent diets to a greater extent than schools, such as the local food environment and parental attitudes towards food.

“I just don’t think it’s an easy fix to get them to make that choice when for us as an organisation we are… where we are our schools are surrounded by fast food, chicken and chips for £1, so it’s difficult.” ID11.

“It’s hard, especially in an area like this, like I said a lot of them are not sitting down and eating, they are used to going to the chippy and it is really hard.” ID12.

Sub-theme 4b: Constraints on the ability of caterers to intervene

Catering managers working within external providers were also constrained by restrictions around suppliers, or use of centralised menus and recipes, limiting their autonomy to introduce the proposed strategies.

For others, there were features of the secondary school setting that were thought to inhibit the implementation of some strategies, seen to be largely out of the control of caterers, such as a lack of physical space (in kitchens and food service areas) and the short duration of lunch breaks. This created a barrier for strategies that require additional space or preparation time (e.g. pre-chopped fruit) or slowed down service (e.g. labelling, self-service).

“We had a salad bar, the kids they haven’t got enough time, the kids don’t get through quick enough, they stopped it.” ID4.

“But the fact that we only have two serving points it means that we’re quite limited in the options that we do, so it would be nice if we could have more options, but right now that’s impossible really” ID3.

Some caterers also felt that food and drink provision was already healthy and balanced in their schools, partly due to the implementation of the national school food standards, so there was perception that there is a limit to the implementation of strategies for items that are already restricted e.g. sugar sweetened drinks, fried foods.

“Yeah, but like I said to be fair our menus are planned out so they are balanced meals anyway, so there’s nothing fried on our counter, and there’s no greasy food or fatty food, so it is a better choice, it is a healthier option.” ID1.

Sub-theme 4c: Adolescent resistance

There were also some beliefs relating to adolescent resistance to healthy eating efforts, and that it would take time for some strategies to be effective as students got used to the changes.

“…because they’re [students] creatures of habit aren’t they? Well we all are, you will just tend to go in and buy the same thing every time, and so maybe if the catering team, if the kitchen is experiencing quite a lot of wastage they will get rid of products when actually maybe sticking with them for a bit longer…” ID5.

#### Enablers

***Theme 6: Perceived role***

Caterers and schools believed that promoting healthy eating is part of their role, so they appeared motivated to intervene. Some catering managers described this as a moral obligation (n = 3), placing pupil health and welfare above profit motives.

“As a school we actually don’t look to make a profit from students. We just want… we always go in with a mind that this could… for some students this is probably one of their most… probably the only main meal they may get through… I am not saying for all students, but that’s what we go in mind, we just want them to have a healthy balanced diet, and it be affordable.” ID8.

Caterers described putting this into practice through various existing strategies, using verbal prompts for pupils to try new dishes or select fruit or vegetable items, which participants described as impactful in influencing selection of these items, although there was some scepticism about whether this influences pupils’ actual consumption.

“Once they walk away that is a hard one, you don’t know if they are going to eat it, so I would like to… in that sense I think it would be nice to know that the message that we’re trying to get over to them they are understanding” ID8.

This perceived role went beyond food provision and extended to social learning opportunities associated with sit-down meals, such as developing table manners and etiquette around eating with a group.

“I think sitting down and having a proper plate and a bowl with your pudding in, and a cup with your water in or whatever in primary, and in secondary, cups with water in, it just looks nicer, it looks like they are sitting down to a proper meal, so just teaches them a bit better manners I think, table manners.” ID11.

***Theme 7: Opportunities***

Sub-theme 7a: Early intervention

Catering teams felt that early adolescence was the key point in which to intervene, with some additional opportunities at sixth form, as adolescents are transitioning to adulthood.

“So I am hoping now we’re working on the lower end of the school then hopefully that will… it will make it a lot easier once over the next 18 months, then we should have the whole school then by that time eating a lot healthier, that’s the plan.” ID1.

Sub-theme 7b: Whole-school approaches

There was also a sense that wider school approaches, and support from school leadership, could support healthy eating and the efforts of catering teams, e.g. having restrictions on foods and drinks brought into school could support the canteen by reducing the competition from packed lunches.

“Packed lunches filled with goodies and chocolate and stuff is likely to be quite appetising to students, so if you’ve got a school that has a healthy packed lunch policy that bans those things it helps us, because suddenly they are not something to aspire to with other students, but actually they look more towards the lunch service options.” ID5.

Sub-theme 7c: Adaptability and responsiveness

Caterers emphasised the need for tailored approaches that suited the pupil population and the specific school context and leadership, in designing appealing menus and selecting healthy food cues. This was reinforced by the idea that some healthy food cues have limited longevity in influencing dietary behaviours, and so a dynamic approach is needed to ensure the intervention retains its novelty and impact.

“I also looked at a study where all the sandwiches that had salads in they put a smiley face on, and then they had a look at the uptake of the sandwiches. I do think it would work, I don’t know whether it would be a short-term thing, something like the stickies in my head would be more of a short-term thing, it would be a novel, I want a sandwich with a sticker on it. But then I don’t know whether it would fizzle out later on.” ID5.

Responsiveness to changing circumstances was also seen to be important in supporting healthy eating. For example, restrictions introduced during the Covid-19 pandemic offered an opportunity to try new ways of operating. The move to more outdoor eating meant that staff adapted products to be more portable and discovered packaging solutions which have allowed main meals to be served in a ‘grab-and-go’ format. Many schools had introduced staggered lunch times to maintain school ‘bubbles’ [groups of pupils], a change that appeared to suit catering teams as the overall duration of lunchtimes increased so it was felt to be less of a rush, and it seemed to help with managing behaviour during lunchtime too.

“We do serve the boxes already in the takeaway pots, and our dishes, some of our curry and rice dishes, things like season things and stuff like that, they are in takeout cartons… and then also our salads are in takeaway boxes, our sandwiches, baguettes, a panini, so they are all in takeaway… which the children like and especially more so since Covid really” ID11.

“Before the Covid restrictions we have one lunch that lasted 40 minutes, and we could get the whole school through. So that was pretty tight” ID10.

On the other hand, participants felt that COVID-19 -related restrictions did limit schools’ ability to implement some strategies to encourage healthy eating (e.g. self-serve salad) as well as opportunities for student engagement.

## Discussion

### Key findings and relationship to other research

This study aimed to collect data from school catering teams to inform the development of future food choice interventions. School caterers reported that CA is suitable in this setting, they gave recommendations for strategies and suggested student engagement in any intervention design. They also highlighted barriers (other drivers of catering decisions and limits of the influence of school catering on student diets) and facilitators (believing promotion of healthy eating to be part of their role and that adolescence is a critical intervention point).

This research suggests that school caterers support the idea of CA strategies in this setting, as they felt it was suited to the population group and considered encouragement of healthy eating an important part of their role. This is consistent with public perceptions of less intrusive interventions (e.g. guiding or enabling choice) as more highly acceptable than intrusive approaches (e.g. eliminating/restricting choice) [39]. Strategies that have salience with catering teams include those that enhance the visual appeal, placement and portability of healthy food items. This suggests that strategies with a behavioural orientation (so-called ‘convenience enhancements’, seeking to affect what consumers do, without necessarily changing their knowledge or emotions) [40] may be particularly acceptable in this setting, as well as those focused on the presentation of the product and wider environments [23]. Pricing and promotion strategies that focused on quantity were also felt to have the potential to guide healthier selection amongst secondary school students. Improving the dining environment was viewed as a means of increasing school food uptake/meal participation, making the service more financially viable.

These findings align with those of other qualitative research with pupils and secondary school staff, which highlight food placement strategies, pricing strategies focused on value-for-money, enhancing visual appeal and increasing portability of healthy items as potentially important strategies in this setting [28, 29, 41–44]. Support for placement/convenience strategies also comes from intervention studies, which have shown such strategies to be effective in increasing vegetable consumption [17] and purchases of fruit, as well as reductions in sweet baked goods and sugar sweetened beverages [45].

Student engagement in the design of menu items and introduction of healthy food choice cues was seen as essential to participants in the current study. Similar to our findings, a systematic review of food service interventions in secondary schools also identified student engagement as a key component of effective interventions [46]. Co-design of interventions with students may enhance the transparency and legitimacy of CA interventions, factors seen to be influential in acceptance of nudge-based approaches [47, 48].

Our study also identified strategies that catering teams thought would be less feasible. Caterers suggested that there would be practical barriers to introducing labelling, due to the recent reduction in the sales of pre-packaged foods in schools and the additional burden that such a strategy would create, as a result of allergy labelling requirements. Labelling was also viewed as impractical due to the additional time that students would require to process the information, which was considered incompatible with the need to move students through the food service area quickly. However, these findings conflict with those of Devine et al. [41] who also conducted qualitative research in UK secondary schools in a similar time period, and found that labelling strategies would be suitable for these settings. The general lack of consensus on this point suggests any labelling that was implemented should seek wide consultation during its design and development. In research on such ‘cognitively oriented’ nudges, descriptive labelling (e.g. energy) was seen to be less effective than evaluative labels (e.g. symbols), with the prior requiring more deliberative cognitive processing than the latter [49]. If labelling is to be implemented in school settings, use of semiotics e.g. emoticons, may have greater acceptability than descriptive labels, as they would overcome concerns around slowing down food service.

Potential barriers to implementing food choice cues to increase healthy food selection in this setting included concerns over loss of custom and increased waste, both of which would impact upon income and therefore financial viability of the service. Such concerns have also been highlighted in other qualitative research [13, 41]. Collecting data on footfall, profits and waste in evaluations, would therefore be required to demonstrate to school caterers whether an intervention to promote healthy diets did not have unintended consequences in these areas of concern. In addition, some caterers wanted to understand the impact of healthy eating cues upon pupil consumption, as well as selection. A systematic review of school meal nudge interventions indicated positive effects upon selection of target foods/drinks, but inconsistent effects upon consumption [17].

This research also highlights that interventions targeted at improving healthy selections in secondary school canteens also need to be considered within the wider context of the school and the external environment, including the local food environment and the home. This suggests that school-based CA interventions cannot be expected to have a large impact upon dietary intakes in isolation and need to be considered as just one component of a broader strategy for supporting healthy eating in adolescents both within school (e.g. alongside whole-school approaches and policies) and beyond the school environment. Devine et al. [28] also found that whole-school initiatives were an important element of future schools-based dietary interventions for adolescents. However, our findings also suggest that wider food environment interventions (such as limiting marketing of unhealthy food to children) may also be required as part of a wider strategy. In addition, our findings support the use of adaptable, responsive approaches to implementing food choice interventions in a school setting, including tailoring to the specific school context (considering the pupil population, school leadership and existing physical space).

### Strengths and limitations

A key strength of this research is that it adds to a growing literature base on the views of catering representatives [29, 41, 50], the group that would be responsible for delivery of a food choice intervention in secondary school canteens. A systematic review of the impact of secondary school food service interventions upon student food behaviours found that inclusion of key stakeholders such as food service staff was crucial to achieving maximum impact [46]. Another strength was our inclusion of schools with a range of catering provision models and varying levels of FSM eligibility, and inclusion of catering representatives from multiple schools and providers, addressing previous limitations in the UK literature [28, 29] and meeting our aim of recruiting at least 10 schools/providers to the study.

However, the sample size was small, driven by the financial limitations of the study and low response rate from invited schools. It may be the case that those caterers responding were more interested in health promotion, and so the findings relating to the perceived role of catering teams should be interpreted with caution. Recruitment from a larger number of schools and providers, and consideration of other school characteristics such as high ethnic diversity or rural location, may have offered additional insights to inform the design of a future intervention that has broad reach. A further limitation to this research was that we considered hypothetical implementation of food choice cues. Although this is useful in considering the design of interventions prior to delivery, qualitative data collection post-intervention to explore experiences of implementing such strategies would provide valuable information on their feasibility and acceptability. Finally, as we used a constructivist approach in this research, the findings are rooted in the interpretations of the researchers. Most of the researchers involved (MM, MP, OO) had prior experience of research on school food, and two of the researchers (MM, AC) had professional backgrounds in nutrition. Given the team’s experience, we may have had some underlying assumptions or pre-formed ideas about the acceptability/feasibility of the strategies featured, and this may have influenced the findings.

### Implications

Guidance on designing complex interventions highlights the importance of considering diverse stakeholder perspectives and considering feasibility and acceptability of interventions [15]. The current study provides insights into the design of a potential future intervention using healthy food cues in secondary schools from the perspectives of catering teams. Incorporating strategies relating to placement/convenience, presentation and pricing/promotion may improve the acceptability of such interventions to catering teams. Although practical constraints suggest lower acceptability and feasibility of cognitively orientated strategies, such as labelling, this does not rule-out their potential use. However, careful consideration should be given to the labelling type (i.e. use of semiotics as opposed to descriptive labelling) and the potential additional practical support and guidance required to address concerns around the additional work labelling may create for catering teams. Our research adds to existing literature that has highlighted the importance of collaboration and engagement with canteen staff in food service intervention design [31, 45, 46]. Likewise, our research supports the engagement of students, with caterers highly supportive of a particpatory/co-design approach. Consultation with key stakeholders, as well as comprehensive review of the prevailing CA in each setting is therefore likely to support the design of the most appropriate intervention for each school, an approach supported by the WHO policy brief on the use of nudges in schools to promote healthy eating [26].

Through exploring the perspectives of caterers, this research also highlights some beliefs that may support or hinder the successful implementation of a food choice intervention. Incorporating existing frameworks, such as the Theoretical Domains Framework [51], to assess potential implementation problems and support intervention design, may be helpful. For example, an intervention may need to include components that address caterers’ beliefs around their limited capabilities in implementing healthy food choice cues; potential restrictions relating to the environmental context and resources (e.g. physical space); and perceptions around the limited role that such an intervention could play. Future interventions could be framed to garner the greatest level of support from catering staff, emphasising the supportive beliefs held by caterers, such as their perceived social/professional role and motivations in supporting healthy eating in secondary school pupils, and views on the importance of maintaining and supporting informed choice in this age group. Presenting a rationale for what additional impact the food choice intervention could have alongside existing strategies/frameworks to support healthy eating in schools e.g. school food standards, may also achieve greater support. In terms of evaluation, the current study suggests that metrics relating to selection, consumption and waste of target foods/drinks, and potential impacts upon footfall/school meal participation and income, are particularly important to catering teams.

## Conclusion

This research contributes valuable insights for informing the design of future food choice interventions in secondary school canteens, by identifying convenience enhancements and presentation strategies as highly acceptable within catering teams, who would bear the main responsibility for the implementation of these strategies. Use of pricing/promotion strategies and tailored, participatory/co-design approaches also had high levels of support. We have identified practical barriers to implementing some strategies, such as the additional work involved in implementing labelling formats and space and time constraints within the secondary school setting. Future interventions could be framed to garner the greatest level of support from catering staff, for example, thinking about how caterers see their role, beliefs about the potential impact of a school canteen intervention upon adolescent dietary intake and views on the importance of maintaining and supporting informed choice in this age group. Evaluation of future interventions should consider potential impacts upon purchasing and consumption behaviours, food waste and school meal participation, to address caterers concerns about these issues.

### Electronic supplementary material

Below is the link to the electronic supplementary material.

Supplementary Material 1

## Data Availability

The datasets generated and/or analysed during the current study are not publicly available but are available from the corresponding author on reasonable request.

## Abbreviations

CA: Choice architecture
COVID-19: Coronavirus disease
FSM: Free school meals
SSB: Sugar sweetened beverage
UK: United Kingdom
US: United States
