# Supplementary material for: Changing the food environment in secondary school canteens to promote healthy dietary choices: a qualitative study with school caterers
Source: BMC Public Health. 2024 Jul 23;24:1970. doi: 10.1186/s12889-024-19513-7 (PMC11267781; doi:10.1186/s12889-024-19513-7)
Supplement: Supplementary file 1 — Supplementary Material 1 [file 12889_2024_19513_MOESM1_ESM.docx]

Topic guide

**Prior to starting interview:**

- Ask if the interviewee has had a chance to read the participant information sheet
- Give a short summary of the research
- Ask if the interviewee has any questions
- Inform the interviewee the interview will be audio-recorded and confirm they are happy to go ahead
- Ensure that the consent form has been completed accurately and collected
- Ensure that the staff demographic questions have been completed

**Questions**

**School food**

1. What influences the food and drinks you serve/provide? / What are the important factors when designing your menu? How are the choices made?
2. What does a ‘healthy school lunch’ mean to you?
3. How do you see your role in relation to ensuring healthy food/drinks are provided in school?
4. What would you change about school food in your school?

**Eating environment**

1. Can you tell me about the lunchtime experience in your school?
2. What are your views on the physical environment for eating at your school?
3. Who is responsible for creating / maintaining the eating environment?
4. How do you think the dining room environment influences food choice of pupils?

**Opportunities/barriers**

1. How easy or difficult is it to support pupils to have a healthy diet?
2. Can you tell me about the costs related to school food? *[Highlight factors discussed earlier such as providing healthy choices; providing quick/easy options; providing ‘fashionable’/popular options etc.]*
3. How do you decide how much to charge the pupils?
4. How do you measure school food provision/do you have targets to meet?

**Attitudes and perceptions of potential nudge strategies**

*Verbal description of nudge [researcher]*

1. How do you feel about this approach to changing food behaviours?

I will read out a list of strategies and would like to get your opinion on them.

1. For each strategy I would like you to think about the following:
2. Do you think this would support pupils to make a healthy lunch selection?
3. How easy would this be to achieve? Why?
4. Who would be involved in implementing this?
5. How expensive would this be to implement?

*Go through the strategies one-by-one and prompt the interviewee to give their view.*

**General questions on these strategies:**

1. Which ones do you like? / Why is this your top selection?
2. Which ones do you dislike? / Why is this your bottom selection?
3. Which ones would be the easiest to implement?
4. Which ones would be impossible to implement?

**Acceptability of behaviour change targets**

1. Which food behaviours do you think are most important for children to change?

**Measuring selection and consumption**

1. If you were to evaluate the impact of these strategies upon pupil’s food selections, how would you go about measuring that?

*Verbal description of plate waste methodology*

1. How easy / difficult would it be to collect data on plate waste?
2. Is there anything else you wish to add that you haven’t already mentioned?

Thank the participant for their time and ensure you have contact details to send a voucher
